# From Joints to the Heart: An Integrated Perspective on Systemic Inflammation

**DOI:** 10.3390/life15040629

**Published:** 2025-04-09

**Authors:** Diana Elena Cosău, Irina Iuliana Costache Enache, Alexandru Dan Costache, Ionuț Tudorancea, Codrina Ancuța, Dragomir Nicolae Șerban, Codruța Minerva Bădescu, Cătălin Loghin, Ionela Lăcrămioara Șerban

**Affiliations:** 1 Faculty of Medicine, “Grigore T. Popa” University of Medicine and Pharmacy, 700115 Iasi, Romania; e_cosau@yahoo.com (D.E.C.); ii.costache@yahoo.com (I.I.C.E.); ionut_tudorancea@yahoo.com (I.T.); codrina_ancuta@yahoo.com (C.A.); dnserban@yahoo.com (D.N.Ș.); minerva.badescu@umfiasi.ro (C.M.B.); ilserban1@yahoo.com (I.L.Ș.); 2 Clinical Rehabilitation Hospital, 700661 Iasi, Romania; 3 “St. Spiridon” Emergency County Hospital, 700111 Iasi, Romania; 4 Department of Internal Medicine, Cardiology Division, University of Texas Health Science Center, Houston, TX 77030, USA; catalin.loghin@uth.tmc.edu

**Keywords:** rheumatoid arthritis, cardiovascular disease, atherosclerosis, systemic inflammation, endothelial dysfunction, pro-inflammatory cytokines, autoantibodies multidisciplinary approach

## Abstract

Background: Rheumatoid arthritis (RA) is an autoimmune inflammatory disease which predominantly affects joints, but it can also lead to significant extra-articular complications, particularly cardiovascular disease (CVD). Chronic systemic inflammation promotes endothelial dysfunction and accelerates atherosclerosis, increasing cardiovascular risk. Methods: Current data were analyzed to explore the mechanisms between RA and CVD, focusing on systemic inflammation, pro-inflammatory cytokine patways (IL-1, IL-6, TNF, and JAK-STAT), and their interactions with traditional cardiovascular risk factors. Recent studies and clinical guidelines were reviewed to highlight gaps and advances in risk assessment and management. Results: Persistent disease activity and the presence of autoantibodies significantly increase cardiovascular risk in RA contributing to atherosclerosis and major cardiovascular events. Data also suggest that anti-inflammatory treatments, including methotrexate and biologic agents, may lower this risk. Conclusion: This review highlights the pathophysiological mechanisms between RA and CVD, and the need for early diagnosis and active monitoring to identify and assess cardiovascular risk. A multidisciplinary approach, involving rheumatologists and cardiologists is essential for optimizing cardiovascular risk management and improving patient outcomes. Optimization of cardiovascular risk management strategies in patients with RA should be an essential component of current medical practice, with the main goal of reducing morbidity and mortality from cardiovascular complications.

## 1. Introduction

### 1.1. Systemic Involvement in Rheumatoid Arthritis

Rheumatoid arthritis (RA) is an autoimmune disease involving a persistent inflammatory condition that causes progressive joint damage, structural deformities, loss of function, and, in extreme cases, death [1,2]. RA has a global prevalence ranging from 0.5% to 2%, with higher incidence observed in smokers, women, and individuals with family cases of rheumatic diseases [2,3].

The chronic inflammation in RA can affect not only the joints, but also lead to extra-articular manifestations including vasculitis and rheumatoid nodules along with cardiovascular, neurological, pulmonary, gastrointestinal, hematologic, and renal disorders [4]. These comorbidities can lead to increased premature mortality and morbidity. It is therefore essential that patients with RA are regularly monitored for associated conditions to prevent and effectively manage severe complications [5].

### 1.2. The Correlation of Rheumatoid Arthritis with Cardiovascular Risks

Studies have shown that RA is associated with a considerably higher risk of mortality and morbidity compared to the general population. Specifically, RA has been found to increase the risk of cardiovascular (CV) mortality by up to 50% in comparison with individuals not afflicted with RA. In addition, CVD is the most common cause of death among people with RA [6]. Retrospective studies have found that the risk of suffering from a coronary syndrome is almost doubled in patients with RA, even after taking risk factors into account [7]. Recent research indicates that the increased cardiovascular risk in patients with RA is like that seen in type 2 diabetes mellitus [8,9].

RA is characterized by a specific pattern of CVD, different from that found in the general population. These can include heart attacks, sudden death, and heart failure (HF) with a preserved ejection fraction, while exhibiting an elevated probability of developing silent myocardial ischemia and unexpected death. Patients are more susceptible to inflammatory processes and the formation of unstable atherosclerotic deposits in coronary arteries [10].

While it is well-known that traditional cardiovascular risk factors such as smoking, high blood pressure, and type 2 diabetes mellitus play a role in increased mortality, they do not entirely explain the increased risk observed among patients suffering from RA [11,12]. Instead, chronic systemic inflammation, specific to RA, is recognized as a major contributor to cardiovascular vulnerability [13,14].

The accelerated progression of atherosclerosis and the exacerbation of adverse cardiovascular risk factor alterations, both traditional and emerging, have been proven as consequences of chronic inflammation in RA [14,15,16,17,18]. More studies suggest that this excessive inflammation contributes to what is known as the “lipid paradox”. This phenomenon reflects an inverse correlation between the lipid profile and CV risk in patients with RA who have not received treatment [19,20]. On the other hand, reducing inflammation with specific RA treatments has been shown to have a positive effect on cardiovascular health [20].

The stages of the atherogenic process range from endothelial dysfunction and thickening of the arterial wall to the formation of atherosclerotic plaques in the carotid arteries, and major events such as non-fatal and fatal myocardial infarction or stroke. Notably, even after considering known CV risk factors, an excessive cardiovascular burden persists. In this context, the guidelines issued by the European League Against Rheumatism (EULAR) underscore the importance of the strict control of disease activity as a pivotal element in reducing cardiovascular risk. These guidelines advocate for annual cardiovascular risk assessment in patients with RA, with supplemental reassessments triggered by modifications in disease-modifying antirheumatic drug therapy (DMARD) [13].

Although these recommendations have helped increase awareness of cardiovascular risk among patients with inflammatory arthritis, evidence suggests that their application is not always consistent and systematic. Furthermore, it has been proposed that these guidelines may underestimate the overall CV risk [6].

RA is linked to common and special risk factors which significantly amplify the chances of developing CVD. Smoking, high blood pressure, and diabetes, along with dyslipidemia, as well as other customary risk factors, contribute to the development of CVD. Nevertheless, RA-specific factors, including chronic inflammation, autoantibodies, along with prolonged disease duration, exacerbate this risk by promoting large systemic inflammation in addition to large vascular damage. Table 1 shows four traditional and four RA-specific risk factors, underscoring the necessity of a customized, multidisciplinary approach to risk assessment and management in RA patients [21].

### 1.3. Scientific Background, Research Gap, and Hypothesis

Despite consistent evidence supporting the association between RA and a significant CVD risk, current risk estimation models do not integrate the pathogenetic particularities of RA, leading to an insufficient and consistently underestimated cardiovascular risk assessment among this patient population. Although this increased susceptibility to CVD has been well documented for several decades, individuals diagnosed with RA receive sub-optimal primary and secondary prevention measures compared to other patients in high-risk groups, thus there is a major unmet need to optimize CVD prevention strategies in this population. Current models of cardiovascular risk estimation focus predominantly on traditional factors such as hypertension, diabetes mellitus, and smoking, neglecting the integration of essential elements such as systemic inflammation, autoimmune mechanisms, disease activity, and rheumatoid arthritis-specific biomarkers. Ultrasonographic assessment of the carotid arteries contributes to a more accurate stratification of cardiovascular risk among patients with RA by detecting atherosclerotic lesions. The EULAR recommendations emphasize the need for annual cardiovascular risk assessment, but their inconsistent application may lead to an underestimation of actual risk. In the United States, guidelines dedicated to cardiovascular risk assessment in patients with rheumatoid arthritis are currently lacking, but the EULAR recommendations provide essential guidance for optimizing cardiovascular risk management in patients with RA [22].

Individuals diagnosed with RA are significantly more likely to develop silent myocardial ischemia with an increased risk of sudden death. These patients are also more predisposed to inflammatory processes and the formation of unstable atherosclerotic plaques in the coronary arteries. In addition, individuals with RA are not only at an increased risk of developing heart failure but also have an increased susceptibility to early death after its onset. At the same time, the inadequate implementation of cardiovascular prevention strategies in patients with RA increases their susceptibility to CVD [23].

The main question of the review is whether the integration of RA-specific factors—such as markers of systemic inflammation, autoimmune status, disease activity, and particular RA biomarkers—can lead to a significant improvement in the accuracy of cardiovascular risk prediction models in this patient population, given the limitations of current models based predominantly on traditional risk factors. It is hypothesized that the integration of RA-specific inflammatory markers, autoantibody profile, and parameters reflecting disease activity into cardiovascular risk prediction models will significantly improve their predictive capacity compared to models based entirely on traditional risk factors. Such a comprehensive approach would allow for more accurate identification of RA patients at increased cardiovascular risk and facilitate the implementation of more effective prevention strategies to reduce cardiovascular complications. Therefore, the main objective of this paper is to provide a detailed analysis of the interplay between RA-specific pathologic mechanisms and CV risk, highlighting the shortcomings of current prediction models based predominantly on traditional risk factors. The article aims to synthesize the latest scientific evidence on the pathophysiological mechanisms by which systemic inflammation, autoimmunity, and disease activity contribute to cardiovascular complications in RA. At the same time, this review aims to investigate the role of integrating RA-specific biomarkers and clinical parameters in improving the accuracy of cardiovascular risk assessment models. The structure of the paper begins with a broad description of cardiovascular manifestations and risk factors associated with RA, continuing with a critical assessment of current prediction models and their limitations. Subsequently, the contributions of imaging and emerging biomarkers in optimizing cardiovascular risk stratification are reviewed.

## 2. Pathophysiological Mechanisms of Rheumatoid Arthritis and Cardiovascular Disease

Increased oxidant stress, local autoimmune responses, and enhanced activity of pro-inflammatory cytokines have all been associated with the development of endothelial dysfunction, earlier onset of atherosclerosis, and a higher predisposition to arrhythmias and other cardiovascular outcomes among patients with RA [24].

### 2.1. Chronic Inflammation and Endothelial Dysfunction

Endothelial dysfunction (ED) represents one of the initial pathological alterations in the artery wall, manifesting from the early stages of atherosclerosis. This dysfunction is characterized by a reduction in nitric oxide (NO) synthesis and bioavailability, resulting in diminished vasodilator function and pro-atherogenic properties. ED has been shown to be strongly associated with the most studied cardiovascular risk factors and has been recognized as an independent outcome predictor of future CV events among the general population [25].

### 2.2. The Association Between Chronic Inflammation and CVD

CVD serves as a compelling example of how systemic inflammation associated with RA can affect organ systems beyond the musculoskeletal system. In addition to the already known risk factors, the chronic inflammation associated with RA, as evidenced through elevated inflammatory markers, autoantibodies, and cytokines, favors the development of multiple comorbidities and contributes to an increased risk of disability and mortality. Atherosclerosis and RA have been observed to share numerous environmental and genetic risk factors, which may contribute to ED. A correlation has been identified between ED and the presence of the common human leukocyte antigen (HLA) epitope DR Beta 1 (DRB1×04) (*p* = 0.01) [13,26]. Polymorphisms located outside the major histocompatibility complex (MHC) domains also seem to be correlated with increased CV event risk independent of traditional risk factors [27,28].

A wide range of inflammatory markers have been associated with increased cardiovascular risk, encompassing C-reactive protein (CRP) levels, the erythrocyte sedimentation rate (ESR), the number of affected joints, and disease activity and severity scores. Elevated serum levels of ACPA and RF characteristics for RA are strongly correlated with CVD risk. The role of circulating immune complexes (CICs) in the pathogenesis of vasculitis is significant, with RF playing a pivotal part. The presence of ACPA has also been found to be linked to a more severe form of RA and to extra-articular manifestations (EMs). A recent study has shown that ACPA-positive patients with non-inflammatory arthritis have a comorbidity profile comparable to that of early-stage inflammatory patients. These findings suggest that the occurrence of comorbidities in ACPA-positive patients may precede any clinical onset of inflammation [5].

ACPA has been linked to a higher risk of CVD and the early development of atherosclerosis. The synovitis-specific inflammatory cellular infiltrates show numerous parallels with the inflammatory processes implicated in arterial wall damage and atherosclerotic plaque formation. Pro-inflammatory cytokines, enzymes that damage the extracellular matrix, and molecules mediating these processes have been identified as factors responsible for both atherosclerotic vascular lesions and synovitis [29].

The endothelium has a fundamental involvement in this pathology, producing vasoactive chemicals that regulate vascular tone and affect the balance between circulating blood cells and the vascular wall. Among the vasoactive substances identified in a study conducted by Reriani et al. (2010) are NO (a vasoactive substance) and other inflammatory markers [30].

Inflammation has been demonstrated to disrupt the equilibrium between NO production and other vasoactive compounds, resulting in ED and, thus, the progression of atherosclerosis [30].

Systemic inflammation plays a central role in all stages of the atherosclerotic process, from the activation of endothelial cells and the trapping of inflammatory cells in the arterial wall, to the differentiation of monocytes and their transformation into foam cells, thus favoring the formation and progression of atherosclerotic plaques. There is compelling evidence to support the direct involvement of these inflammatory mediators in atherogenic mechanisms by enhancing endothelial dysfunction, inducing oxidative stress on the vascular layer, facilitating foam cell accumulation, and promoting atherosclerotic plaque instability [31].

Cytokines like TNF-α, IL-17, IL-6, and interleukin-1 beta (IL-1β) are frequently identified in both CVD and RA patients. These inflammatory molecules involved in the pathogenesis of the synovial pannus, are also linked to endothelial cell activation, thereby triggering atherosclerotic processes. Furthermore, they have been shown to amplify the process of coagulation cascades within blood vessels and contribute to increased susceptibility to atherosclerotic plaque rupture [5].

### 2.3. Molecular and Inflammatory Mechanisms

Cytokines have been the focus of extensive research as potential therapeutic targets in RA because of their direct implication in the pathogenesis disorder. These molecules are classified into two main categories—pro-inflammatory and anti-inflammatory—according to the specific roles they play in modulating the immune response to antigens [32].

**Figure 1:**
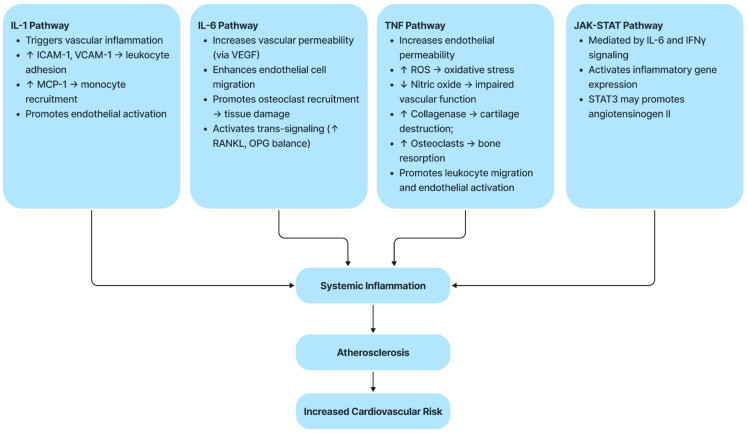
Pathophysiological link between chronic inflammation and CV risk in RA [35]. ↑—increase; ↓—decrease.

Pro-inflammatory cytokines, which include, IL-1β, TNF-α, IL-7, IL-6, IL-17, IL-15, IL-23, IL-18, interferon-gamma (IFN-γ), and granulocyte-macrophage colony-stimulating factor (GM-CSF), play a key role in the modulation of the inflammation process implicated in the mechanisms of RA which are summarized in Figure 1. Elevated levels of these cytokines were detected in synovial tissue, synovial fluid, serum, and peripheral blood of patients of RA. Furthermore, IL-15, IL-17, GM-CSF, and Il-23 have been shown to have a significant correlation with the presence of RF, seropositivity of IL-23, ACPA positivity, and disease activity, suggesting their potential as biomarkers for RA diagnosis [29]. IL-7 is also considered a possible biomarker for early diagnosis of RA, with significantly different levels in the initial stages of the disease [33,34].

The pathogenesis of RA and its cardiovascular complications are deeply influenced by several pro-inflammatory pathways. These molecular mechanisms not only sustain joint inflammatory processes, but also favor endothelial dysfunction, the development of atherosclerosis, and the amplification of cardiovascular risk. Table 2 summarizes the main inflammatory mediators involved and their contribution to cardiovascular pathogenesis.

### 2.4. Brief Description of Each Pathway

#### 2.4.1. The IL-1 Pathway

The IL-1 family includes 11 different types of interleukins. The term IL-1 is commonly used to refer to both IL-1α and IL-1β, essential pro-inflammatory cytokines that are key to inflammasome activation and signaling. Their effects are counteracted by IL-1 receptor antagonist (IL-1Rα), an endogenous inhibitor that blocks their interactions with specific receptors, thereby limiting inflammatory responses [35].

IL-1β promotes the production of adhesion molecules, such as intercellular adhesion molecule-1 (ICAM-1), and chemokines, in particular monocyte chemoattractant protein-1 (MCP-1), that attract inflammatory cells to blood vessel walls [35]. Increased IL-1 levels can trigger an acute phase reaction, amplifying inflammation and atherosclerosis progression [36].

Certain matrix metalloproteinases (MMPs), such as MMP-1, MMP-13, and MMP-8, can degrade the fibrotic structure of the atherosclerotic plaque, increasing its vulnerability to damage and the formation of thrombi [33,35]. In advanced plaques, IL-1β inhibition has been shown to stimulate the release of IL-10, a target cytokine involved in the beneficial remodeling [36,37].

These discoveries led to clinical trials of IL-1 blockade in patients with acute myocardial infarction [35].

Recent studies have shown that targeting IL-1β with drugs like canakinumab reduces the risk of cardiovascular events in patients who have had a heart attack. This supports the use of IL-1β inhibitors as a possible treatment for atherosclerosis [31].

#### 2.4.2. The IL-6 Pathway

IL-6 is a pleiotropic cytokine with regulatory roles in immunity, hematopoiesis, and inflammation [34]. In vascular biology, IL-6 is produced by cells involved in atherothrombotic processes, such as fibroblasts, macrophages, endothelial cells, and monocytes [35,38,39]

This cytokine has three major signal transduction pathways. In classical (cis) signaling, IL-6 attaches to its membrane receptor (IL-6R), forming a binding complex with the glycoprotein 130 (gp130) transmembrane protein which initiates intracellular signaling. In the second pathway, called trans-signaling, IL-6 binds to a soluble IL-6 receptor (sIL-6R), which together with gp130 triggers signaling. The third mechanism, known as trans-signaling, requires interaction between a dendritic cell and a T-cell to facilitate specific signaling [35].

In patients with RA, higher levels of IL-6 and its soluble form, sIL-6R, are associated with stronger synovial inflammation [39]. IL-6 contributes to joint damage and inflammation by increasing vascular permeability and endothelial cell migration, a process related to vascular endothelial growth factor (VEGF) [37,39]. Furthermore, IL-6 promotes osteoclast recruitment, enhancing bone degradation and cartilage destruction [39]. Two approved treatments, Sarilumab and Tocilizumab, utilize monoclonal antibodies, blocking both sIL-6R receptors and membrane-bound receptors [45,46]. IL-6 is correlated with an elevated cardiovascular risk in RA patients, regardless of the presence of traditional risk factors [47,48].

The 2017 Canakinumab Anti-inflammatory Thrombosis Outcome Study (CANTOS) trial investigated the effects of IL-1α inhibition on inflammation. It revealed that reduced IL-6 levels correlated with a lower risk of cardiovascular outcomes [49]. Additional CANTOS data analysis demonstrated a link between lowered IL-6 levels and a reduced risk of cardiovascular events. Those with no significant decrease in IL-6 levels after treatment with Canakinumab had rates of cardiovascular events similar to the placebo group. Increases in IL-6 levels after a heart attack have been seen as potentially contributing to impaired myocardial remodeling and the destabilization of atherosclerotic plaques [50].

#### 2.4.3. The TNF Pathway

TNF is a cytokine with multiple biological functions which exerts its effects through two distinct receptors: TNF receptor 2 (TNFR2) and TNF receptor 1 (TNFR1). While TNFR1 is widely expressed, TNFR2 is highly restricted and is predominantly localized to specific immune cells [51]. The activation of TNFR1 leads to inflammation, whereas TNFR2 controls immune responses [52]. TNF is a key cytokine in the management of RA, as its overproduction can trigger a form of erosive inflammatory arthritis while its inhibition can be effective in treating it [53,54,55,56].

In the synovial tissue of patients with RA, TNF promotes synoviocytes and cartilage degradation through the stimulation of collagenase synthesis. TNF also contributes to bone resorption, osteoclastogenesis, and joint destruction [57,58,59]. The blockade of TNF has been shown to rapidly lower IL-6 levels and acute phase reactants, inhibiting leukocyte migration and endothelial cell activation [60,61].

Current evidence on the link between the ASCVD risk and TNF pathway is limited [62,63]. TNF exerts effects on the endothelium by reducing NO bioavailability, producing of reactive oxygen species (ROS), and augmenting endothelial permeability. TNF has also been identified in human atherosclerotic plaques [40]. Studies in experimental atherosclerosis models have shown that suppression of the TNF gene can slow disease progression, indicating a possible proatherogenic role for this cytokine. However, findings from other mouse models have been inconsistent. Large observational studies have shown a correlation between elevated plasma levels of TNF and a higher risk of recurrence of cardiovascular events [64,65].

The best-documented efficacy of TNF inhibition in cardiovascular disease is its impact on HF [63,65]. In animal models, TNF has been linked to left ventricular systolic dysfunction. However, trials on TNF inhibitors like Infliximab and Etanercept for systolic HF showed no benefit regarding rehospitalization or mortality rates. A subset of subjects who were given Infliximab 10 mg/kg had a higher hospitalization rate than the placebo group [66,67]. This evidence indicates that global TNF inhibition may result in a disruption of both detrimental and protective signaling pathways within HF patients, so this approach is usually avoided in systolic cardiac dysfunction. However, these studies have focused on the general population and not on patients with RA, where TNF is a key pathogenic factor [68].

#### 2.4.4. The JAK–STAT Pathway

Recent advancements have identified the Janus tyrosine kinase/signal transducers and activators of transcription (JAK-STAT) pathway as a promising therapeutic target of RA. Current studies are investigating the link between JAK inhibition and cardiovascular incidents [69]. This pathway consists of four JAK proteins (JAK1, JAK2, JAK3, and TYK2) and seven related STAT proteins. The pathway is triggered by cytokines binding to their receptors, resulting in JAK protein phosphorylation, STAT protein recruitment, and subsequent activation. Activated STAT proteins then move to the nucleus of the cell and attach to specific regions of deoxyribonucleic acid (DNA), thereby stimulating the transcription of designated target genes [70]. This plays a critical role in the immune cell proliferation cellular processes, including apoptosis [71,72].

The JAK-STAT pathway is important in the development of atherosclerosis by modulating IL-6 and TNF. IL-6 activates this pathway by binding to a specific receptor [41]. IFN-γ also contributes to the progression of atherosclerosis by activating JAK1 and JAK2 [44].

Not all JAK-STAT-regulated processes are pro-inflammatory. For instance, STAT3 has been observed to stimulate the synthesis of angiotensinogen II in certain myocardial cells. However, it has also been observed to have a cardioprotective effect in other cardiac cells [42,43]. Future research is needed to clarify the role of JAK inhibition in the development of cardiovascular disease and the potential applications this drug class might hold in future treatments [36].

## 3. Clinical Cardiovascular Manifestations in RA

Cardiovascular manifestations in RA are highly polymorphic, affecting various structures of the cardiovascular system and frequently causing diagnostic problems. Nonspecific symptoms and an overlap with systemic inflammation make the identification of these complications difficult, underlining the importance of careful monitoring and early intervention to reduce cardiovascular risk [43].

### 3.1. Pericarditis

Pericarditis, the most common heart condition associated with RA, often develops during the first stages of the disease and can sometimes appear even before RA is diagnosed [43].

Although less than 15% of rheumatoid arthritis patients show clinically noticeable symptoms, electrocardiograms (ECGs) have shown that between 20% and 50% of them suffer from pericardial involvement. This is often manifested by chest pain or the shortness of breath. On the other hand, cases in which audible pericardial friction rubbing noises, accompanied by hemodynamic disturbances caused by pericardial disease, are uncommon and affect less than 10% of RA patients [73,74].

Autopsies show that between 20% and 40% of RF seropositive patients have pericarditis, although clinical signs are evident in less than 10% of severe RA cases. Pericarditis often occurs in men with destructive forms of RA, a feature common to other EM of the disease. Earlier diagnosis of pericarditis is essential to improve the prognosis of patients with RA. In this regard, careful physical examination and tests for antibodies, such as antinuclear antibodies, RF, and ACPA, are indispensable, especially in the presence of pericardial effusion. In most cases, pericarditis develops after the beginning of arthritis but can occasionally precede the RA diagnosis. Although rare, severe forms, such as rapidly progressing effusive or constrictive pericarditis carry high risks of morbidity and mortality. Treatment of RA-associated pericarditis includes non-steroidal anti-inflammatory drugs (NSAIDs), corticosteroids, or immunosuppressive drugs. Pericardiectomy may be necessary in severe cases. Early intervention and appropriate disease management are vital to prevent complications and enhance the affected patients’ quality of life [73,75].

### 3.2. Sudden Cardiac Death (SCD)

Patients with RA exhibit a 1.6- to 2.4-fold increased risk of SCD compared to the general population, a phenomenon largely attributed to a higher incidence of malignant arrhythmias, even in the absence of traditional cardiovascular risk factors. Chronic systemic inflammation plays a significant role in arrhythmogenesis, contributing directly by influencing cardiac electrophysiology, or indirectly by increasing the progression of cardiovascular structural disease [76].

Inflammation in RA is associated with cardiac dysfunctions like prolongation of the QTc interval, QT dispersion, and dysregulation of the autonomic nervous system, manifested by an increased sympathetic tone and reduced parasympathetic activity. These dysfunctions contribute to electrical instability, which increases the risk of life-threatening ventricular arrhythmias. Importantly, such an arrhythmic risk may exist even in patients without evident structural heart disease [71,73,77].

Pro-inflammatory cytokines, especially TNF-α, have been shown to modulate the functioning of ion channels, leading to a prolonged action potential duration and increased repolarization heterogeneity. Electrocardiographic markers like QTc prolongation and QT dispersion serve as clinical indicators of this electrical instability. A large prospective study demonstrated that a prolonged QTc interval is an independent predictor of mortality in RA with a strong correlation to elevated CRP levels, reinforcing the inflammatory basis of the arrhythmic risk [78].

Autonomic imbalance, shown by decreased heart rate variability, has also been consistently reported in RA. This reflects increased sympathetic activity, which contributes further to arrhythmia susceptibility and cardiovascular events, including SCD, even in the absence of overt structural myocardial damage [78].

These insights emphasize the importance of early detection of subclinical conduction abnormalities—preferably via ambulatory ECG monitoring—and tight control of RA disease activity. Anti-inflammatory therapies such as DMARDs and biologics have shown potential not only in improving joint-related symptoms but also in normalizing electrophysiological parameters and reducing cardiovascular mortality. Randomized trials are warranted to confirm their effectiveness in lowering SCD risk in this vulnerable population [4,78].

RA patients have twice the risk of HF than the overall population, even when cardiovascular risk predictors such as ischemic heart disease (IHD) are considered. This condition is more prevalent among women with RA and is one of the leading causes of mortality among this population. At onset, HF is often preceded by diastolic dysfunction, present in about 66% of patients developing RA. In many cases, these patients maintain a preserved ejection fraction (>50%), but clinical symptoms may be silent, which may complicate both diagnosis and treatment [22,79,80]. The progression of HF in RA is linked to increased levels of inflammatory biomarkers such as the erythrocyte sedimentation rate (ESR), CRP, ACPA, RF, and inflammatory cytokines [81]. Also, the disease activity score-28 (DAS-28), used to assess the severity of RA, is an important indicator of the risk of HF in patients with this condition [82]. RA patients have an abnormal ECG morphology associated with left ventricular diastolic dysfunction, suggesting an increased predisposition to developing HF [80].

In a cohort study of 2045 adults diagnosed with RA, with a mean age of 60.3 ± 13.9 years and a female predominance of 57.6%, a significant association between congestive HF and overall and cardiovascular mortality was identified. During a median follow-up of 109 months, 602 deaths occurred. After adjusting for confounders, patients with congestive HF were found to have a 60% higher risk of all-cause death and a 110% higher risk of cardiovascular mortality compared to those without congestive HF [83].

This association was particularly evident among women and patients over 65 years of age, whereas no significant association between congestive HF and oncologic mortality was identified. The results indicate that the presence of congestive HF in patients with RA is associated with a substantial increase in the risk of death, both overall and from cardiovascular causes. This emphasizes the need for rigorous monitoring and early therapeutic interventions in this vulnerable group of patients [84].

Recent findings emphasize that HF in RA is not only a consequence of traditional cardiovascular comorbidities, but also reflects pathophysiologic mechanisms specific to this condition, fueled by inflammatory cytokines such as TNF, IL-1, and IL-6 involved in rheumatoid synovitis and joint destruction. This persistent inflammation contributes to myocardial remodeling and fibrotic development and is considered a key factor in the development of HF, particularly in the context of RA [85].

Studies using echocardiography and cardiac magnetic resonance imaging (MRI) show that RA patients have subclinical diastolic dysfunction, even in the absence of HF. This is marked by increased myocardial stiffening and impaired relaxation. These changes have been documented in case–control studies comparing patients with RA to those without HF. Echocardiography and MRI parameters reflecting myocardial inflammation and fibrosis are also correlated with RA activity. This suggests that the inflammatory process affecting myocardial structure may begin long before the onset of overt symptoms [85,86].

Autoantibodies and disease activity scores linked to RA are associated with HF, according to the Mantel et al. study. The non-ischemic form of HF is linked to ESR values above 40 mm/h and a DAS28 score above 5.1. The Mayo Clinic has also shown increased cardiovascular risk in patients with RF, elevated ESR, and extra-articular manifestations, suggesting that immuno-inflammatory processes may damage the heart in patients with RA. Therefore, screening for CVD is paramount, even in patients with no clinical manifestations [85].

### 3.3. Cardiomyopathy

Cardiomyopathy is a group of conditions, usually without a known cause, that directly involve the heart muscle and do not have ischemic, congenital, hypertensive, valvular, or pericardial causes. There are two systems for classifying cardiomyopathies, one based on functionality (hypertrophic, dilated, and restrictive) and primary/secondary classification, where RA is among the etiologies. In RA, cardiomyopathy may result from processes such as non-specific focal myocarditis, diffuse necrosis, or granulomatous myocarditis, which are histologically diagnosed. Postmortem studies have reported the presence of these lesions in approximately 3–30% of RA patients [43,74,82].

Interleukin-1α (IL-1α) and other inflammatory mediators, together with cell fragments from the damaged myocardium, trigger the activation of inflammasomes in various cells, thus contributing to the inflammatory response [43,84]. This IL-1α-mediated uncontrolled inflammatory process contributes to the loss of myocardial contractile tissue, cardiomyocyte apoptosis, and the development of fibrosis [43,87]. In addition, persistent inflammation associated with active RA influences the long-term systolic function of the heart and molecular remodeling. These changes favor the progression of heart failure in patients with rheumatic disease [78]. The use of certain medications for RA, such as corticosteroids and methotrexate, has also been associated with the development of cardiomyopathy, which complicates the determination of the exact etiology [43,74,88].

### 3.4. Coronary Artery Disease (CAD)

RA patients are at a significantly higher risk of developing myocardial infarction and atherosclerotic CAD, reported since the 1960s, with an autopsy detection rate of 20%. The chronic inflammation in RA accelerates the progression of atherosclerosis through endothelial dysfunction and dyslipidemia, characterized by decreased high-density lipoprotein (HDL) cholesterol and increased low-density lipoprotein (LDL) cholesterol. RA and atherosclerosis share common autoinflammatory mechanisms, including the involvement of inflammatory cytokines and similar genetic factors, which increase the susceptibility of RA patients to develop CAD. CAD symptoms are more severe in these patients with RA, being associated with unstable plaques, elevated coronary calcium scores, and multivessel damage. CV prevention strategies recommended for the overall population should also be implemented among patients with RA to reduce the risk of CV complications [43,89,90].

Data from a large population-based cohort in Sweden showed a reduction in mortality among patients with RA; however, the risk of death remains significantly increased in people with CAD and RA, which supports the findings of this review. Furthermore, a meta-analysis of 24 observational studies with a total of 111,758 subjects reported 22,927 deaths from cardiovascular causes, showing a 50% increase in mortality risk in patients diagnosed with RA [91].

Cardiac amyloidosis, a rare cause of restrictive cardiomyopathy, is characterized by the infiltration of the myocardium with fibrillar proteins, leading to a loss of compliance and impaired diastolic and sometimes systolic function. Although definitive diagnosis is based on histologic analysis, ultrasonography of the heart may reveal a “sparkling” pattern, and ultrasonography combined with MRI may reveal biventricular hypertrophy. The presence of cardiac amyloidosis in RA is not well documented and is considered a rare complication. Case-series studies have reported a variable incidence, possibly influenced by patient selection. The condition is more common in men with a prolonged duration of disease and has a major clinical relevance considering its association with heart failure, a frequent cause of morbidity in RA patients on hemodialysis. If amyloidosis is diagnosed in RA patients, intensified immunosuppressive therapy should be considered to reduce disease progression [74].

### 3.5. Rheumatoid Nodules

Rheumatoid nodules, also called rheumatoid granulomas, can affect multiple structures of the body, including the epicardium, epicardial fat, interventricular septum, myocardium, chordae tendineae, heart valves, and aorta. Their presence in cardiac structures can lead to functional complications such as arrhythmia or valvular damage. Symptoms associated with the presence of rheumatoid nodules are rare, but may include syncope or even death caused by a heart blockage due to a localized lesion in the conduction system. Nodules located on heart valves may also lead to stroke or other manifestations of arterial embolisms [74,92]. Although immunosuppressive treatments are fundamental for the management of RA, there is no evidence to show that they can eliminate rheumatoid nodules located in the heart. In cases where nodules cause significant cardiac dysfunction, drug interventions or surgical treatments may be necessary [74].

### 3.6. Arrhythmias

Arrhythmias are one of the leading causes of mortality in RA and are frequently associated with ischemia, conduction abnormalities caused by amyloidosis, rheumatoid nodules, or congestive HF. The increased sympathetic activity seen in RA patients may also contribute to tachyarrhythmias [74,93].

Atrial fibrillation is the most common arrhythmia in patients diagnosed with RA and is seen predominantly in women under 50 years of age, especially in the presence of ESR > 60 mm/h or anti-TNF-α antibodies. A cohort study conducted in Denmark, which included 4,182,335 participants (18,247 of whom were RA patients), reported an incidence of AF approximately 40% higher compared to the general population [94].

However, a subsequent meta-analysis including three retrospective cohort studies showed a pooled relative risk of developing atrial fibrillation of only 1.29 among RA patients compared to the control group. This small difference is because, in two of these studies, the increased risk of atrial fibrillation was no longer significant after adjusting for comorbidities and treatments given. In addition to atrial fibrillation, other types of arrhythmias are commonly identified in RA, although reported in smaller cohort studies, including premature ventricular contractions and ventricular tachycardia [94].

Although modern treatments and early diagnosis have improved survival in RA patients, cardiovascular risk remains significant. RA is associated with accelerated endothelial dysfunction, arterial stiffening, and premature atherosclerosis, which contribute to a risk of SCD twice that of the normal population. This risk is independent of conventional risk factors. Corrected QT intervals (QTcs) and QT dispersion (QTd), indicators of abnormal ventricular repolarization, are longer in RA patients than in healthy controls. This suggests that QTd might be a valuable marker for assessing the risk of cardiovascular mortality and morbidity associated with complex ventricular arrhythmias. Furthermore, disease duration seems to influence this parameter. In long-term RA patients with moderate disease activity, QTc prolongation does not differ significantly from that observed in the normal population with similar cardiovascular risk factors. However, QTd is considerably more common in RA patients. These results indicate that maintaining disease activity at a moderate level in the long term may help lower the risk of ventricular arrhythmias in RA. However, the residual risk associated with QTd prolongation remains present and requires careful monitoring [74,95].

### 3.7. Valvular Diseases

**Figure 2:**
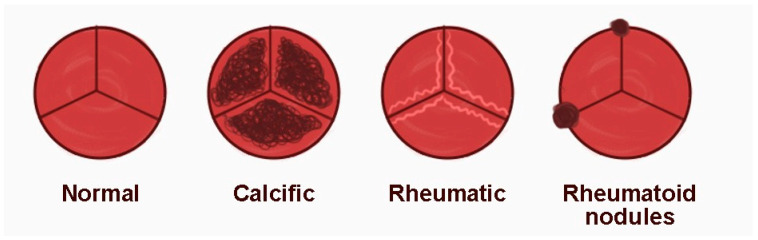
Spectrum of aortic valve disorders and the typical aspect of rheumatoid nodules [96].

In RA, the heart valves most affected are the mitral and aortic valves, and the valvular damage usually occurs after joint manifestations. The lesions are generally focal and asymptomatic, rarely causing significant valvular dysfunction, although cases of severe aortic regurgitation have been reported. Studies show RA patients have a four times higher risk of the thickening and calcification of the aortic valve and twice the risk of aortic regurgitation than the general population. Rheumatoid nodules localized on valves can be differentiated from vegetations by localization and mobility. Vegetations usually occur on the cusp tips on the low-pressure side of the valve and are characterized by independent mobility. In contrast, rheumatoid nodules are located on the medial portion and base of the cusp and are immobile (Figure 2). These nodules are thought to arise due to vascular trauma or valvular fibrosis generated by extra-articular inflammation. Valvular pathology in RA may include myxoid degeneration, fibrosis, fusion of valve cusps, and chronic inflammation, which plays a key role in progressive valve damage. In rare cases, rheumatoid nodules may cause significant cardiac dysfunction, arrhythmias, or embolic events, in which case invasive intervention may be necessary. Currently, there are no specific treatments for asymptomatic rheumatoid cardiac nodules, and their management depends on their impact on the cardiac function [96].

### 3.8. The Role of Cartilage Oligomeric Matrix Protein (COMP) in Rheumatoid Arthritis Pathogenesis

COMP, also recognized as thrombospondin 5, is a marker of cartilage turnover, normally localized in synovial joints but also present in arterial walls. It is present in higher amounts in atherosclerotic plaques, particularly those with vulnerable features. Circulating COMP, used as a marker of cartilage activity in RA, has been correlated to a higher risk of atherosclerotic CAD and CVD. Increased risk predictors include persistent systemic inflammation, high DAS28 scores two years after diagnosis, and cumulative disease activity. These associations are unrelated to conventional cardiovascular risk factors. Awareness of elevated cardiovascular risk in patients with difficult-to-treat RA is essential to improve prognosis and reduce cardiovascular complications. Baseline serum COMP levels may provide valuable prognostic information for identifying patients at higher risk of developing CAD and CVD, emphasizing the importance of monitoring this marker in clinical practice [97].

## 4. Diagnosis and Monitoring

### 4.1. Inflammatory and Metabolic Markers

#### 4.1.1. Classic Inflammatory Markers

##### C-Reactive Protein (CRP)

CRP is an independent marker of cardiovascular (CV) risk in both the general population and in patients with RA. CRP levels above 3.0 mg/L are associated with a 58% higher risk of CAD in the normal population compared to values below 1.0 mg/L. In RA, elevated CRP levels are correlated with an increased risk of cardiovascular incidents, particularly HF, myocardial infarction, CV mortality, and stroke, increasing by 14% for each mg/L of CRP. Elevated CRP levels are also associated with the development of subclinical atherosclerosis, as measured by CMIT. CRP contributes to the promotion of atherosclerosis by direct pathways involving activation of the complement system, induction of apoptosis, and ED. The systemic inflammation associated with RA intensifies the process and CRP plays a significant role by recruiting monocytes, increasing the inflammatory response and promoting atherosclerotic plaque instability. It also favors thrombus formation through platelet activation [98].

##### Specific Markers in RA and CVD

Adiponectin, leptin, and IL-6 are key biomarkers of CV risk in RA, having a close association with inflammation and metabolism. Adiponectin is related to a higher risk of HF and mortality, particularly in non-obese individuals, and leptin and IL-6 reflect the link with non-traditional and traditional risk predictors, emphasizing the importance of managing inflammation to reduce CV risk [99,100].

##### Interleukin-6

IL 6 has been associated with dyslipidemia, due to its role as an inducer of the acute inflammatory phase, which affects the lipid profile. Increased concentrations of IL-6 contribute to hypertriglyceridemia and increased LDL cholesterol levels by inhibiting hepatic lipoprotein lipase activity, stimulating triglyceride synthesis, and enhancing lipolysis in adipose tissue. Furthermore, IL-6 correlates with CRP levels, with the ability to stimulate CRP production in hepatocytes. A positive association has also found between cardiovascular risk, IL-6 levels, and disease activity [99].

#### 4.1.2. Vascular Biomarkers (Leptin, Adiponectin)

##### Leptin

Leptin is an adipokine with pro-inflammatory as well as pro-atherogenic effects, thus it is closely linked to CV risk. A Swedish study in the general population showed significantly higher plasma leptin levels among patients with acute myocardial infarction, emphasizing the role of leptin as a key factor in obesity-associated CV risk. The correlation between the positivity of RF and CV risk was also observed, attributed to the influence of RF on lipoprotein metabolism, with a predominance of atherogenic lipoproteins. Research has shown higher concentrations of leptin in RF-positive compared to RF-negative patients, although some studies have reported conflicting results, probably due to differences in sample levels and RF frequency. Leptin is also positively associated with RA disease activity. Meta-analyses have demonstrated significant associations between leptin concentrations and DAS-28 CRP scores, reinforcing its biomarker role for disease activity. Additionally, leptin levels are associated with metabolic syndrome and a higher body mass index (BMI), reflecting the involvement of adipose tissue in inflammatory processes and endothelial damage [99].

##### Adiponectin

Adiponectin has been identified as significant regarding CV risk among RA patients. Elevated adiponectin levels have been linked to a significantly higher risk of hospitalization due to HF and CV mortality, especially in non-obese individuals. These findings suggest that adiponectin may indicate the presence and severity of chronic disease, being useful in predicting hospitalization, and morbidity. However, it does not appear to be a specific marker for acute events such as myocardial infarction, stroke, or venous thromboembolism (VTE) [100].

Although adiponectin is generally recognized as an anti-inflammatory molecule, its effects may vary depending on the context and tissue type. In RA-affected chondrocytes it has been found to activate the VEGF and other inflammatory mediators, indicating potential pro-inflammatory effects. Elevated adiponectin levels could represent compensatory mechanisms in patients with vascular injury, chronic inflammation, or cachexia, including cachexia associated with RA or HF [100].

Research has also shown a link between disease activity and elevated adiponectin levels in RA, particularly in low body weight patients. This association could be explained by interactions between weight loss, inflammatory cytokines, and increased adiponectin secretion. Also, high adiponectin levels have been correlated with markers of RA severity, such as osteoporosis and radiographic lesions, suggesting a possible role of this molecule in disease progression [100].

Although adiponectin shows potential as a predictor of hospitalization HF and CVD mortality, its effect is more evident among non-obese patients. This emphasizes the need for further research to better understand the role of adiponectin in maintaining cardiovascular health and its impact on inflammatory conditions like HF and RA [100].

### 4.2. Cardiac Specific Biomarkers

Cardiac biomarkers play an essential role in the assessment and prediction of CVD risk in patients with RA. NT-proBNP (N-terminal pro-B-type natriuretic peptide) has been shown to be a relevant marker of ventricular dysfunction and cardiac ischemia, being frequently elevated in RA patients, despite the lack of overt CVD risk factors. This biomarker reflects compensatory ventricular responses to pressure and volume overload and is a reliable predictor of major adverse cardiovascular events (MACE). High-sensitivity troponin T (hsTnT) is also strongly associated with subclinical myocardial injury and microvascular dysfunction. Unlike NT-proBNP, hsTnT correlates more directly with cardiac structure and function, indicating the presence of myocardial stress and myocardial ischemia. Similarly, high-sensitivity troponin I (hsTnI) has been identified at elevated levels among patients with RA, suggesting subclinical myocardial injury that is independent of inflammatory markers or traditional cardiovascular risk factors. Another valuable biomarker is anti-Apo lipoprotein A-I (anti-Apo A-I) immunoglobulin G (IgG) antibodies, which are linked to the presence of atherosclerotic plaques and are an indicator of MACE risk. Combined with conventional cardiovascular risk measurement models, for example, the Framingham risk score (FRS), these biomarkers contribute to a more accurate prediction of cardiovascular events. In addition, oxidized LDL (ox-LDL), released from inflamed joints of RA patients, plays a key role in the process of atherogenesis. Elevated levels of ox-LDL are correlated with both disease activity and the presence of subclinical atherosclerosis, highlighting the link between chronic inflammation and cardiovascular risk. Therefore, biomarkers such as NT-proBNP, hsTnT, hsTnI, anti-Apo A-I, and ox-LDL contribute significantly to the early identification of cardiovascular abnormalities and accurate cardiovascular risk stratification in RA patients. Their integration into clinical practice may optimize the decision process and improve patient prognosis [23].

Table 3 highlights key cardiac biomarkers linked to cardiovascular risks in RA. These biomarkers, such as NT-proBNP, hsTnT, anti-Apo A-I IgG, and ox-LDL, demonstrate the strong connection between chronic inflammation and cardiovascular disease (CVD). Incorporating these markers into clinical evaluations can support the early detection of subclinical atherosclerosis and myocardial injury while improving the risk assessment for CVD in RA patients [23].

**Figure 3:**
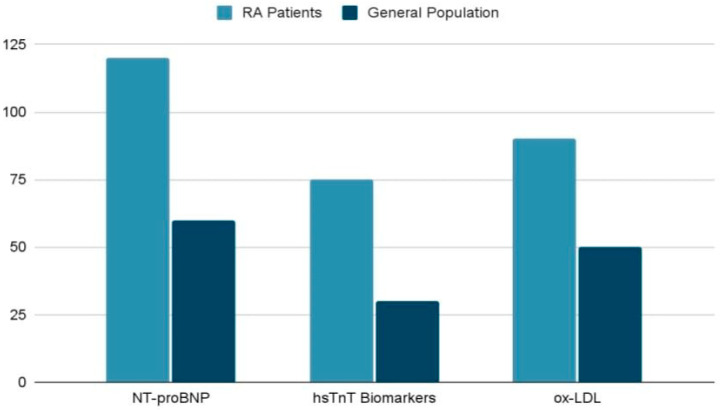
Comparison of biomarkers levels: RA vs. general population [23].

The following diagram (Figure 3) demonstrates the involvement of three cardiovascular biomarkers (NT-proBNP, hsTnT, and ox-LDL) and their impact in RA patients compared to the general population. All of these biomarkers were found to have significantly higher levels in RA patients compared to the general population, demonstrating that the systemic inflammation in RA leads to an increased cardiovascular risk with direct implication on vascular damage. The differences highlighted in the diagram emphasize the influence of RA on CV risk through chronic inflammation and its effects on specific biomarkers. This highlights the need for monitoring and treatment strategies to prevent cardiovascular complications in this patient population [23].

### 4.3. Emerging Biomarkers and Future Directions

Recent research has identified a number of emerging biomarkers that could be effectively used for the early detection of CV risk in patients diagnosed with RA [24,101].

#### 4.3.1. Ischemia Modified Albumin (IMA)

IMA represents a relevant biomarker as it reflects early pathological processes such as endothelial dysfunction and oxidative stress, both of which are involved in the development and progression of atherosclerosis. In a 2024 study by Erre and coworkers, a statistically significant inverse correlation (rho = −0.22, *p* = 0.02) between serum IMA levels and peripheral vasodilatory capacity was reported, thus highlighting the potential of this molecule as an early marker of endothelial damage and subclinical cardiovascular disease in patients with RA. The results of this study indicate that IMA may represent a relevant and promising biomarker for the identification of ED in the context of RA. However, further investigations are needed to validate these observations and to assess the practical value of IMA in the early diagnosis and monitoring of atherosclerotic processes in RA patients [24].

#### 4.3.2. Catestatin (CST)

CST is a peptide resulting from the processing of chromogranin A and plays a key role in modulating cardiovascular functions by reducing catecholamine secretion and stimulating NO synthesis and histamine release. In patients with RA, a significant correlation was observed between elevated levels of CST and the presence of RF and ACPA. These associations suggest that CST could be a biomarker with prognostic value, especially in seropositive RA patients at an increased cardiovascular risk [101].

#### 4.3.3. Fetuin-A

Fetuin-A is a glycoprotein with recognized functions which inhibit inflammation and tissue calcification processes. It was negatively correlated with various inflammatory markers, such as ESR and plasma fibrinogen level and with DAS28-ESR in RA, particularly in women. Sex-stratified analyses revealed that serum fetuin-A values showed a significant inverse correlation with ESR, fibrinogen, and DAS28-ESR. These findings suggest that fetuin-A may have a protective effect in RA-associated cardiovascular pathology, although this connection with surrogate cardiovascular markers requires further investigation to be clearly defined [101].

Although the current results on these biomarkers are promising, their wide clinical application remains limited, mainly due to the lack of validation in large heterogeneous cohort studies. It is essential that future investigations aim to confirm the clinical utility of these biomarkers, further explore the mechanisms by which they correlate with cardiovascular pathology in RA, and integrate them into comprehensive cardiovascular risk assessment algorithms [24,101].

### 4.4. Practical Limitations and Specificity Issues in Biomarker Interpretation

Although cardiac biomarkers are useful tools for identifying and monitoring cardiovascular risk in RA, their clinical interpretation faces significant difficulties due to low specificity and the increased risk of false-positive results. Cardiac biomarkers, such as hsTropT and NT-proBNP, are useful tools in the assessment of structural and functional abnormalities of the heart. However, the interpretation of elevated values of these biomarkers in patients diagnosed with RA is often complicated by the presence of extracardiac factors. For example, elevated hsTropT concentrations, commonly associated with subclinical myocardial involvement, may also be caused by other pathologic conditions such as persistent systemic inflammation or chronic renal dysfunction, which reduce diagnostic accuracy and make it difficult to accurately assess cardiovascular risk in RA patients [23].

In the same way, NT-proBNP, a well-known biomarker for prognostic relevance in cardiac pathologies, may be elevated not only in HF but also in other medical conditions such as pulmonary hypertension, renal failure, or various systemic inflammatory diseases commonly found in RA. Thus, the multitude of factors that may influence these values increases the risk of false-positive interpretations, further complicating the clinical decision-making process and affecting the accuracy of cardiovascular risk stratification in these patients [23].

Although C-reactive protein (CRP) is widely used as a marker of systemic inflammation, its applicability in monitoring RA disease activity and estimating cardiovascular risk is limited by several factors. Data from observational clinical trials indicates that a significant number of patients may have normal CRP values despite overt inflammation, suggesting that this biomarker partially captures the complexity of RA inflammation. Extrinsic factors such as body fat mass, female hormones, diet, or stress might also influence CRP, leading to a misinterpretation of the results. Thus, a low CRP level might create a false impression of controlled inflammation, even in the presence of clinical manifestations. It is also important to note that CRP is not a stand-alone parameter for RA risk or RA diagnosis. CRP use should be accompanied by an integrative clinical analysis and correlated with other biomarkers for an accurate assessment of disease activity and cardiovascular risk [97].

Leptin and interleukin-6 (IL-6) are relevant cardiovascular risk markers in patients with RA, but clinical interpretation is difficult due to low specificity and the influence of confounders. IL-6 values correlate with clinical parameters, like disease activity, elevated triglycerides, LDL-cholesterol, and smoking, emphasizing the association with cardiovascular risk score. However, elevated values may also indicate metabolic or endocrinologic disorders. Gender, BMI, and the presence of metabolic syndrome affect leptin concentrations, and IL-6 may be influenced by immunosuppressive treatments like hydroxychloroquine. Using these biomarkers as single predictors may generate false-positives. Integrated interpretation in a complex, multidimensional clinical setting is recommended [98].

However, the practical application of this method involves some specific difficulties, as biomarker values may vary under the influence of several individual patient characteristics, including age and the therapeutic regimens used, such as non-steroidal anti-inflammatory drugs, glucocorticoids, or disease-modifying anti-rheumatic drugs. At the same time, the interpretation of biomarker data becomes even more complex when associated diseases such as diabetes mellitus or hypertension are present, highlighting the need for detailed and personalized clinical analyses. For this reason, there is a need for further research and development of effective strategies to support the validation and widespread integration of cardiac biomarkers into practice, with the fundamental aim of optimizing the management and improving the prognosis of patients diagnosed with RA [23].

### 4.5. Cardiovascular Risk Assessment in Patients with RA

The assessment of total CV risk is crucial to identify those who may benefit most from preventive interventions. Although RA is an autonomous CVD risk indicator, the presence of RA alone does not automatically justify the use of cardio-preventive medication. Over 360 models have been proposed to predict cardiovascular (CVD) risk, most of which include common risk predictors, for example, lipid profile, blood pressure, age, smoking, and gender. Most of these aim is to predict ACVD, while others extend the analysis to include broader outcomes such as HF and cerebrovascular disease. Key models are illustrated in Table 4 [22].

RA-specific models, such as the ATACC-RA and ERS-RA algorithms, integrate both traditional factors and RA disease-specific features. However, they have shown limited predictive accuracy. On the other hand, tools developed for the general population, such as SCORE and FRS, tend to underestimate cardiovascular risk in RA patients, most likely because of the independent influence of chronic inflammation. These findings emphasize the need to develop better tailored tools for CV risk assessment in the context of RA [22].

Among the existing variants, QRISK3 seems to be the most viable option in practice as it includes RA as an independent risk factor with a specific weighting coefficient and integrates additional information on comorbidities and socio-demographic parameters. However, the attempts to adapt the scores developed for the general population—including QRISK3—have so far not yielded sufficiently accurate predictions for patients with RA. In the absence of extensively validated models, it is recommended to use QRISK3 in combination with the EULAR multiplication factor and, where possible, to complete the assessment with imaging investigations such as carotid ultrasound for a better estimation of individual risk [22].

### 4.6. Integrated Strategies: Collaboration Between Rheumatologists and Cardiologists

Patients with RA are at a significantly higher risk of developing CVD, despite adjustments for traditional risk factors. Absolute CVD risk in people with RA is equivalent to that of individuals without RA, but who are 5–10 years older, and CVD-related mortality and morbidity are comparable to those observed in individuals with type 2 diabetes mellitus. However, risk scores based on traditional factors alone frequently underestimate the true risk, particularly in women with RA. To address this limitation, the EULAR suggests adjusting standard CVD risk scores by multiplying by a factor of 1.5 in patients with inflammatory arthritis, although this method requires further validation. Inflammation is a major factor in increasing CV risk in RA patients, interacting with traditional risk predictors in a complex way that is not yet fully understood. The contribution of inflammation is not sufficiently incorporated by current risk assessment models, such as those including hs-CRP [102].

Inflammation control, especially through therapies such as Methotrexate, has been demonstrated to be successful in lowering CV death in RA patients. Biological agents are also being investigated for their potential benefits on cardiovascular health. These findings highlight the need for integrated approaches targeting both inflammation and traditional risk factors to effectively reduce the risk of CVD in RA patients. Close collaboration between rheumatologists and cardiologists is crucial for the optimal treatment of these patients. In addition to aggressively targeting rheumatic disease to reduce inflammation, it is essential to detect and manage traditional cardiovascular risk predictors. Whereas conventional methods of cardiovascular risk evaluation tend to underestimate the risk in RA patients, innovative tests such as CIMT, a sensitive marker of atherosclerosis, may provide valuable insights. Studies have shown an increase in the value of CIMT in RA patients compared to healthy individuals, but its predictive value for future clinical events requires further research. Cardiovascular risk assessment RA patients focus on identifying risk factors, treating traditional cardiovascular risks, and using therapies such as methotrexate and low-dose corticosteroids. Close collaboration between rheumatologists and cardiologists is vital to improve patient care [102].

Specialized clinics, such as Preventive CardioRheuma in Norway and CardioRheum at the Mayo Clinic in the US, highlight the importance of a multidisciplinary approach. These centers prioritize patient and medical staff education, complex cardiovascular risk assessments using non-invasive and invasive methods, and personalized plans that consider both traditional risk factors and the specifics of RA disease. They also include research programs dedicated to the continuous improvement of prevention strategies [101].

There is clear evidence that people with RA and related autoimmune diseases have a significantly higher cardiovascular risk compared to the general population. This underscores the need for improved efforts to recognize and modify risk factors, detect disease early, and implement effective treatment strategies for this unique group of patients. Promisingly, innovative multidisciplinary care models are being developed to address this critical need [102].

## 5. Future Directions

Despite the significant progress made in elucidating the association between RA and CVD, several critical questions remain unanswered. Future research directions should focus on improving cardiovascular risk assessment models by integrating inflammatory and immunologic markers specific to RA pathology. Furthermore, it is imperative to validate emerging biomarkers such as CST, fetuin-A, and IMA in large prospective studies to determine their real clinical applicability.

In addition, experimental investigations are needed to clarify the direct effects of chronic systemic inflammation on myocardial repolarization, autonomic nervous system dysfunction, and the cardiac remodeling process. Last but not least, it is essential to conduct controlled clinical trials to comparatively assess the impact of anti-inflammatory therapies—including biologic agents and JAK inhibitors—on cardiovascular risk reduction in RA.

The adoption of integrated multidisciplinary models of care, bringing together rheumatologic and cardiologic expertise, is vital for translating these scientific advances into concrete clinical benefits for RA patients.

## 6. Conclusions

AR is strongly correlated with a significantly increased CVD risk as a consequence of persistent systemic inflammation and immune dysfunction. This review highlights the main mechanisms linking RA and CVD, including endothelial dysfunction, pro-inflammatory cytokine pathways (IL-1, IL-6, TNF, and JAK-STAT), and the presence of conventional cardiovascular risk factors. Existing data emphasize the importance for early diagnosis, constant monitoring, and intensive RA management to limit associated cardiovascular risks. Incorporating cardiovascular screening into routine rheumatologic care, refining risk assessment tools, and personalizing therapeutic strategies based on activity and comorbidities are key priorities. Future research should explore targeted interventions that address joint and vascular inflammation to improve patient care.

## Figures and Tables

**Table 1:** CV risk factors in RA patients [21].

Category	Specific Risk Factors	Key Points	Reference
Traditional Risk Factors			Yu et al., 2023 [21]
Smoking	Doubles CVD risk in RA.	Encourages smoking cessation interventions.	
Hypertension	Affects ~70% of RA patients; often underdiagnosed.	Screening every 5 years recommended.	
Diabetes	Correlated with a twofold increase in CVD risk in RA.	Anti-diabetic therapies that minimize inflammation.	
Dyslipidemia	Paradoxical ↓ low-density lipoprotein (LDL) levels ↑ CV risk.	Lipid-monitoring statins.	
RA-Specific Risk Factors			
Chronic inflammation	High sensitivity C-reactive protein test (hsCRP) and Interleukin-6 (IL-6) highlight systemic inflammation.	Methotrexate, tumor necrosis factor (TNF) inhibitors ↓ CVD risk.	
Disease activity and duration	Longer disease activity ↑ CVD risk.	Aggressive RA control is vital.	
Use of glucocorticoids	Prolonged use risk ↑ risk of hypertension, diabetes, dyslipidemia.	Dose minimization recommended.	
Autoantibodies-Rheumatoid factor (RF), Anti-Citrullinated Peptide Antibody (ACPA)	RF-positive are 2.5 times more at risk of HF.	Requires tailored monitoring and therapy.	
Novel Risk Factors			
Carotid intima–media thickness (CIMT)	Elevated in RA patients.	Non-invasive atherosclerosis marker for early CVD detection.	
Valvular disease	↑ Prevalence; linked to systemic Inflammation.	Echocardiography for monitoring.	

↑—increase; ↓—decrease.

**Table 2:** Key inflammatory pathways involved in RA and their cardiovascular impact [35,36,37,38,39,40,41,42,43].

No.	Inflammatory Marker	Pathogenic Mechanism	References
1	IL-1β	Stimulates intercellular adhesion molecule-1 (ICAM-1), matrix metalloproteinases (MMPs) activation	Weber et al., 2023 [35]
2	IL-6	Promotes vascular inflammation, vascular endothelial growth factor (VEGF)-mediated permeability	Libby et al., 2013 [37] Srirangan et al., 2010 [39]
3	TNF-α	Promotes endothelial dysfunction, reactive oxygen species (ROS) production, and increased permeability	Hansson et al., 2006 [40]
4	IFN-γ	Activates JAK-STAT pathway, enhances inflammatory response	Ivashkiv et al., 2018 [44]
5	STAT3	Dual role: pro-cardiac hypertrophy (via angiotensinogen II)/cardioprotection (via IL-10)	Krishnamurthy et al., 2009 [42] Plens-Galaska et al., 2018 [43]

**Table 3:** Biomarkers associated with CV risk in RA [23].

Biomarker	Association	Clinical Role	Reference
NT-proBNP	Ventricular dysfunction	MACE	Borra et al., 2023 [23]
hsTnT	Myocardial injury	Detects subclinical cardiac damage	
Anti-Apo A-I IgG	Atherosclerotic plaque presence	FRS predictive accuracy	
ox-LDL	Atherogenesis, inflammation	Indicates disease activity	

**Table 4:** Comparison of CV risk models and their limitations in RA [22].

Risk Model	Target Population	Key Features	Limitations	Reference
SCORE (Systematic Coronary Risk Evaluation)	General population (Europe)	10-year CVD mortality risk.	Fatal events only; no RA-specific adjustments.	Semb et al., 2020 [22]
FRS	General population (USA)	Includes stroke, vascular events, HF.	Overestimates risk; based on predominantly white cohorts.	
American College of Cardiology (ACC)/American Heart Association (AHA) Pooled Cohort Equation (PCE)	General population (USA)	Considers ethnicity, 10-year ASCVD risk.	Not RA-specific; traditional risk factors only.	
QRISK3	General population (UK)	Includes RA (weight = 1.4); integrates multiple risk factors.	May overestimate risk; limited validation outside the UK.	
Reynolds Risk Score (RRS)	General population (USA)	Incorporates hsCRP for improved risk estimation.	Not validated for RA; may underestimate risk.	
ERS-RA (Expanded Risk Score for RA)	RA-specific	Combines traditional with RA-specific characteristics.	Limited validation: modest predictive accuracy.	
ATACC-RA (Transatlantic Cardiovascular Risk in RA)	RA-specific	Integrates RA-specific inflammation markers with traditional factors.	Requires further validation, inconsistent results.	

## Abbreviations

The following abbreviations are used in this manuscript:

ACCA	American College of Cardiology
ACPA	Anti-Citrullinated Peptide Antibody
AHA	American Heart Association
ASCVD	Atherosclerotic cardiovascular disease
ATACC-RA	Transatlantic Cardiovascular Risk in Rheumatoid Arthritis
BMI	Body mass index
CANTOS	Canakinumab Anti-inflammatory Thrombosis Outcome Study
CAD	coronary artery disease
CHD	coronary heart disease
CICs	Circulating immune complexes
CIMT	Carotid intima–media thickness
COMP	Cartilage oligomeric matrix protein
CRP	C-reactive protein
CV	Cardiovascular
CVD	cardiovascular disease
DAS-28	Disease activity score-28
DMARDs	Disease-modifying antirheumatic drug therapy
DNA	Deoxyribonucleic acid
DRB1	DR Beta 1
ECG	Electrocardiogram
ED	Endothelial dysfunction
EMs	Extra-articular manifestations
ERS-RA	Expanded Risk Score for RA
ESR	Erythrocyte sedimentation rate
EULAR	European League Against Rheumatism
FRS	The Framingham Risk Score
GM-CSF	Granulocyte-macrophage colony-stimulating factor
Gp130	Glycoprotein 130
HDL	High-density lipoprotein
HF	Heart failure
HLA	Human leukocyte antigen
hsCRP	High-sensitivity C-reactive protein test
hsTnI	High-sensitivity troponin I
hsTnT	High-sensitivity troponin T
ICAM	Intercellular adhesion molecule
IFN-γ	Interferon-gamma
IHD	ischemic heart disease
IgG	Immunoglobulin G
IL	Interleukin
IL-1β	Interleukin-1 beta
IMA	Ischemia-modified albumin
JAK/STAT	Janus tyrosine kinase/signal transducers and activators of transcription
LDL	Low-density lipoprotein
LDL-C	Low-density lipoprotein cholesterol
MACE	Major adverse cardiovascular events
MCP	Monocyte chemoattractant protein
MHC	Major histocompatibility complex
MMPs	Matrix metalloproteinase
MRI	Magnetic Resonance Imaging
NO	Nitric oxide
NSAIDs	non-steroidal anti-inflammatory drugs
NT-proBNP	N-terminal pro-B-type natriuretic peptide
OPG	Osteoprotegerin
ox-LDL	Oxidized LDL
PCE	Pooled Cohort Equation
QTc	Corrected QT interval
RA	Rheumatoid arthritis
RANKL	Receptor activator of nuclear factor kappa-Β ligand
RF	Rheumatoid factor
RRS	Reynolds Risk Score
ROS	Reactive oxygen species
SCD	Sudden Cardiac Death
SCORE	Systematic Coronary Risk Evaluation
sIL-6R	Soluble IL-6 receptor
TNF	Tumor necrosis factor
TNF-α	Tumor necrosis factor alpha
TNFR1	TNF receptor 1
TNFR2	TNF receptor 2
VEGF	Vascular endothelial growth factor
VTE	Venous thromboembolism
